# Turning the Tide: A 2°C Increase in Heat Tolerance Can Halve Climate Change‐Induced Losses in Four Cold‐Adapted Kelp Species

**DOI:** 10.1002/ece3.71271

**Published:** 2025-04-24

**Authors:** Griffin Hill, Clément Gauci, Jorge Assis, Alexander Jueterbock

**Affiliations:** ^1^ Faculty of Biosciences and Aquaculture Nord University Bodø Norway; ^2^ CCMAR University of Algarve Faro Portugal

**Keywords:** assisted evolution, climate change, heat tolerance, kelp forests, restoration, species distribution models

## Abstract

Kelp forests are susceptible to climate change, as their sessile nature and low dispersal capacity hinder tracking of suitable conditions. The emergence of a wide array of approaches to increasing thermal tolerance seeks to change the outlook of biodiversity in a changing climate but lacks clear targets of impactful thermal resilience. Here, we utilize species distribution models (SDMs) to evaluate the potential of enhanced thermal tolerance to buffer the effects of climate change on cold‐adapted kelp species: *Saccharina latissima*, *Alaria esculenta* , *Laminaria hyperborea* , and *Laminaria digitata* . For each species, we compared a baseline model—where the thermal niche remained unchanged—to models where the simulated maximum sea surface temperature tolerance was increased by 1°C–5°C. These models were projected into three climate change scenarios: sustainability (Shared Socioeconomic Pathway (SSP) 1‐1.9, Paris Agreement), regional rivalry (SSP3‐7.0), and fossil‐fuel development (SSP 5‐8.5). Our SDMs demonstrate that an increase of 1°C–2°C in thermal tolerance could recover over 50% of predicted losses of suitable habitat for cold‐adapted kelps. However, *A. esculenta, a species of growing commercial interest,* still faced persistent habitat contraction across all climate change scenarios and simulated tolerance increases, including up to 15% unrecovered losses under SSP5‐8.5, even with a simulated 5°C increase in thermal tolerance. Our findings highlight the need for a two‐pronged approach to conserve cold‐adapted kelp forests: stringent reductions in greenhouse gas emission reductions in line with the SSP1‐1.9 scenario, and strategies to boost kelp's thermal tolerance by at least 1°C–2°C. This dual approach is crucial to maintain 90% of the current suitable habitat of *S. latissima* and *L. digitata* , and 70% for *A. esculenta* and *L. hyperborea* . Relying on mitigation or adaptation alone will likely be insufficient to maintain their historic range under projected climate change.

## Graphical abstract

Our model findings highlight the need for a two‐pronged approach to conserve cold‐adapted kelp forests: stringent reductions in greenhouse gas emissions in line with the SSP1‐1.9 scenario, and strategies to boost kelp thermal tolerance by at least 1°C–2°C. This dual approach is crucial to maintain 90% of the current suitable habitat of S. latissima and *Laminaria digitata* , and 70% for *Alaria esculenta* and *Laminaria hyperborea* .

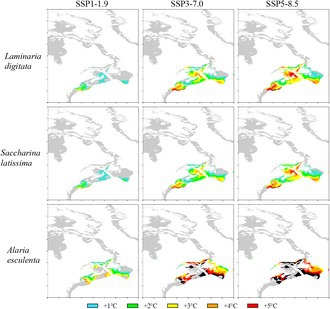

## 1 Introduction

Kelps (brown algae of the order of the Laminariales) are foundational species of one of the most diverse and productive ecosystems on earth, the kelp forests (Mann 1973). They provide a direct habitat for a wide variety of invertebrates but also serve as refuge and nursery for fish and foraging area for predators, making a link between lower and higher trophic levels (Teagle et al. 2017). However, kelp forests have been declining, in part due to climate change, at the unprecedented pace of 2% per year globally, which is twice as fast as reported coral reef decline and four times faster than rates seen in terrestrial tropical forests (Filbee‐Dexter et al. 2022). Kelp forests are particularly threatened in the North Atlantic, where the coverage has decreased by 65% in southern Norway and by 76% on the east coast of North America, mainly attributed to marine heatwaves (Filbee‐Dexter et al. 2020). Some of the most prolific kelp species are cold adapted, with ranges centered on nutrient‐rich, cold temperate waters, making adaptation in a warming climate for these species simultaneously constrained by biology and phylogeny (Muth et al. 2019). Losses are forecast to continue and even intensify in a changing climate, with projected global reductions by up to 15%, while present‐day lower latitude kelp forests are likely to see high turnover and an overall reduction in diversity (Assis et al. 2024). *Laminaria hyperborea* , the most harvested species in the North Atlantic, faces declines of up to 40% and local extinction in the Iberian Peninsula, where it has been largely replaced by the warm‐temperate kelp *Laminaria ochroleuca* (Assis et al. 2016; Barrientos et al. 2025). Thus, global warming compromises both the future sustainability of natural habitats and the production security of associated industries (Jueterbock et al. 2021).

Traditional kelp forest management has primarily relied on passive measures that do not involve direct manipulation of kelp populations, such as the establishment of Marine Protected Areas (MPAs), predator regulation, and harvest restrictions (Eger et al. 2022). While these strategies have helped reduce anthropogenic pressures, they often fail to restore lost kelp populations, and their effectiveness under projected climate change remains uncertain (Eger et al. 2022). Consequently, proactive measures, such as out‐planting of kelp in declining forests, are increasingly employed to protect at‐risk populations. However, simple transplantation may not be sufficient if the transplanted individuals lack the necessary thermal tolerance or adaptive potential to thrive in future conditions (Coleman et al. 2020). To address this, emerging strain enhancement techniques offer tools to boost thermal tolerance but lack concrete targets tied to meaningful increases in survival (Zhou et al. 2022). For each vulnerable population, it is crucial to determine the specific increase in thermal tolerance required to ensure habitat suitability under future climate scenarios to better guide restoration efforts and enhance the long‐term success of these initiatives.

The goal of this study was to investigate the mitigation potential of increased heat tolerance to cope with future climate in the North Atlantic cold‐adapted kelp species *Saccharina latissima*, *Alaria esculenta* , *L. hyperborea* , and *Laminaria digitata* . To do so, we developed species distribution models (SDMs) where for each species, we compared a baseline model—where the thermal niche remained unchanged—to models where the simulated thermal niche was increased by 1°C–5°C. These models were then applied to increasingly severe climate scenarios to assess the impact of a given tolerance increase in recovering suitable habitat area that would otherwise be lost in the base model. We aimed to identify targets of increased tolerance to better guide kelp restoration efforts.

## 2 Materials and Methods

### 2.1 Occurrence Data and Modeling Area

Occurrence data were retrieved from the curated global dataset for marine forest (Assis et al. 2020) and the Ocean Biodiversity Information System (OBIS) via the R package robis (Provoost and Bosch 2017; OBIS: Ocean Biodiversity Information System 2024). Records were filtered to remove duplicates and entries with high spatial uncertainty. Records were confirmed with known scientific literature as well as the AlgaeBase and MarLIN databases (Araújo et al. 2016; Guiry and Guiry 2024; Hiscock and Tyler‐Walters 2006).

To capture the present and possible future distribution of suitable habitat, the study area was delineated to the North Atlantic and adjacent seas (longitude bounds: −85° W, 50° E; latitude bounds: 35° N, 75° N) with a high‐resolution coastal layer restricted to a depth of 200 m due to the potential for future offshore farming beyond where wild populations occur (Tullberg et al. 2022). Habitat suitability was modeled in the whole study area for each species, regardless of the species' actual distribution of occurrences, to include potential suitable habitat (Figures A3 and A4).

### 2.2 Environmental Variables

Environmental variable data was retrieved from Bio‐ORACLE 3.0 via the R package biooracler (Assis et al. 2024). We selected seven environmental variables, based on their relevance in a review of previous modeling efforts focused on macroalgae and climate change, to construct our SDM base model: maximum sea surface temperature (°C, SST), salinity (PSS), nitrate (mol m^−3^), sea ice thickness (m), sea water velocity (ms^−1^), and phosphate concentrations (mol m^−3^) (Fragkopoulou et al. 2022). These environmental layers were created from monthly averages between the period 2000–2020. Sea ice cover was removed because it was highly correlated with maximum SST (threshold > 0.8, Spearman's rank correlation). Of the remaining parameters, nitrate and sea water speed were removed because they made less than a 5% contribution to the model output for all species. Salinity was also removed for *L. digitata* and *L. hyperborea* because it contributed below the 5% threshold. Maximum SST was used to reflect the role of acute warming events, such as marine heatwaves, and summer maximum temperatures in limiting kelp distributions (Gurgel et al. 2020). To further explore the influence of enhanced heat tolerance, we modified the maximum SST values. We used the same variables (maximum sea surface temperature (°C, SST), salinity (PSS, only for *S. latissima* and *A. esculenta* ), and phosphate concentrations (mol m^−3^)) to predict the distribution of our kelp species under scenarios SSP1‐1.9, SSP3‐7.9, and SSP5‐8.5 (Meinshausen et al. 2020). These environmental layers were created from projected monthly averages in the period 2090–2100 for each scenario.

Occurrence records were gridded to the consistent resolution of the Bio‐ORACLE 3.0 environmental layers (0.05° × 0.05°, approximately 5.5 km × 5.5 km grid cells at the equator) (Assis et al. 2024).

### 2.3 Model Building

The SDMs for *S. latissima*, *A. esculenta* , *L. digitata* , and *L. hyperborea* were built using the MaxEnt algorithm via the SDMtune package MaxNet function (MaxEnt without Java) for R. Maxent is an SDM method using presence‐only data (no absences or generated pseudoabsences are required) to estimate the probability distribution of maximum entropy and is well‐suited to the abundance of presence‐only occurrence data for the study species (Phillips and Dudík 2008). We used spatially and environmentally separated training and testing for fold‐based cross‐validation, providing a robust error estimation of our models using the blockCV package (Valavi et al. 2019). Independent blocks were created based on spatial autocorrelation minimization and randomly assigned to one of 5 folds covering the entire modeling region. Each output was then an average of the sub‐models, each corresponding to the unique combinations of training on four folds and testing on the one remaining. We selected a set of hyperparameters (linear, quadratic, and product feature combinations; beta regularization multiplier = 1) that minimized overfitting based on the partial dependency plots and found the best combination based on the difference in area under the curve (AUC) of the receiving operating characteristic (ROC) between train and test datasets.

To compare suitable habitat under the three scenarios and five simulated heat tolerance increases, we binarized the MaxEnt “clog log” output based on a per‐species threshold that captured 95% of the true occurrence cells, and the number of cells corresponding to presence was tallied for each scenario and species. By subtracting the binarized suitability values in the present from the binarized suitability values under the different climate scenarios for each cell, we could estimate the number of cells lost per scenario and species and convert it into lost area. These binarized SDMs represent the fundamental niche of each species as limited by the variables considered.

### 2.4 Heat Tolerance Increase

**FIGURE 1:**
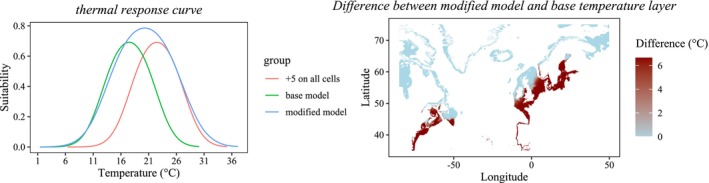
Response curves (left) for the base model (green), +5°C on all cells (red), and modified model (blue, transition function applied). The resulting temperature modification (right) across the modeling region.

To simulate the potential increase in the species heat tolerance by 1°C–5°C, we trained new models with the same hyperparameters and environmental variables but using a transformed temperature layer. A flat 1°C–5°C increase of the max SST for all cells above the species' thermal optimum would create a disjointed response curve, missing temperatures, and model artifacts that are difficult to mitigate. Therefore, we modified the temperature layer in a way that the trained model would produce a response curve that encompasses the response curve of the base model and of a model with the desired tolerance increase on all cells (Figure 1a). To do so, we applied a logistic function to smoothly transition from the normal temperature to the temperature that encompasses both response curves using the formula:

$$y\;=\;\frac{\text{temperature}\;\text{increase}}{\left(1\;+\;e^{\left(-\text{increase}\;\text{rate}\;\times \;x-\text{suitability}\;\text{peak}\right)}\right)}$$

where *y* is the new temperature in each cell; *x* is the unmodified temperature; *temperature increase* is the temperature that encompasses both response curves (species and tolerance increase specific); *increase rate* is the steepness of the transition and represents the number of cells where the temperature is modified (Figure 1b, held constant); *suitability peak* is the optimum temperature of the response curve of the base model. This approach was repeated for all tolerance increases from 1°C to 5°C. Suitability values for each model were binarized, using the same approach as for the base model.

To estimate the reduction in suitable kelp habitat, we subtracted the area of suitable cells (post‐binarization) in the present‐day base model from the projected future binarized area of suitable cells. To assess the potential of enhanced heat tolerance to mitigate future habitat loss, we calculated the “recovered area” for each 1°C increase in thermal tolerance by subtracting the future suitable area of the baseline model (representing no adaptation) from the future suitable area of the corresponding model with enhanced tolerance. We expressed this recovered area as a percentage of projected habitat loss projected for each species and climate scenario, providing a measure of the relative benefit of each 1°C tolerance increase. Scripts and key input files are available for public download, per the Data Accessibility Statement.

## 3 Results

### 3.1 Model Evaluation

All models reported an AUC of the ROC above 0.85 (average across models of 0.88, Table A1), indicating a high performance in predictions. Models had an average True Skill Statistic (TSS) > 0.61 (minimum 0.57, maximum 0.68), indicating substantial discriminating power (ability to correctly classify presence vs. absence) for a prevalence‐independent kappa‐type metric (Allouche et al. 2006; Landis and Koch 1977). *A. esculenta* and *L. hyperborea* exhibited the lowest temperature suitability peak at approximately 15°C, followed by *L. digitata* at 16°C and *S. latissima* at 17°C (Table A1). For all species, temperature was the main environmental driver with more than 40% of contribution (30% for *L. hyperborea* ), reflecting a fundamental niche driven primarily by temperature.

### 3.2 Area Lost Under Future Climate Change

**FIGURE A1:**
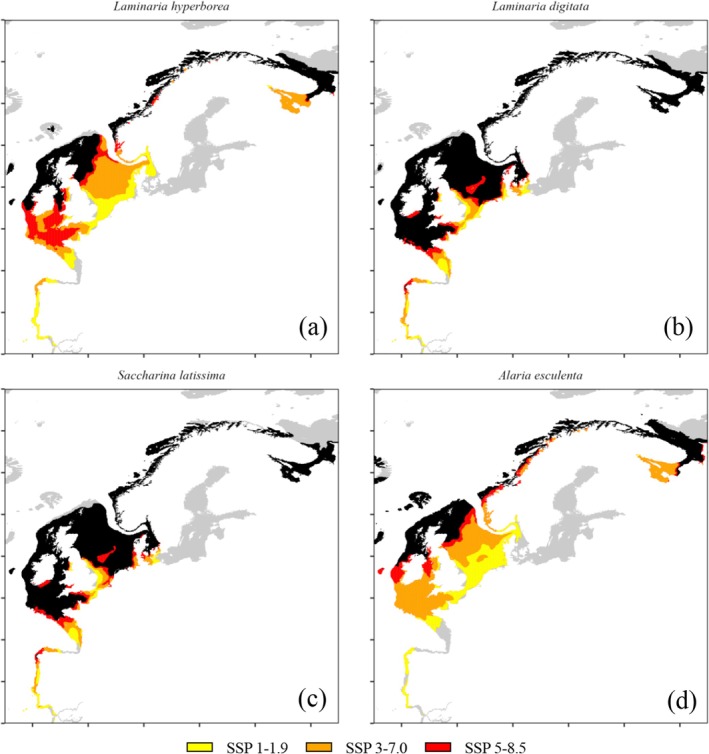
Projected habitat loss in the East Atlantic for *Laminaria hyperborea* (a), *Laminaria digitata* (b), *Saccharina latissima* (c), and *Alaria esculenta* (d) by 2100 under SSP1‐1.9 (yellow), SSP3‐7.0 (orange), and SSP5‐8,5 (red). The areas shown for each scenario are cumulative, meaning they include the losses from the previous, less severe scenario(s). Only the additional area lost under a given scenario is shown in its corresponding color. Areas in gray are modeled, but not suitable (see Figure A3). Areas in black are suitable, but not projected to be lost under the climate change scenarios considered.

On the east side of the Atlantic, all kelp species had projected losses from southern Norway to the south of the Iberian Peninsula (Figure A1). Overall, habitat contraction was lower than on the west side of the Atlantic, capped for SSP5‐8.5 at 54% in *L. hyperborea* and *A. esculenta* and 15% in *L. digitata* and *S. latissima* (Table 1, Table A2). The minimum latitude of projected suitability (Table 1) ranged from 36.8° N (Gibraltar, *L. digitata* , SSP1‐1.9) to 53.5° N (Hamburg, *A. esculenta* , SSP5‐8.5).

**TABLE 1:** Area lost (in 1000 km^2^), habitat contraction (in %) and minimum latitude of suitability (°N) in the East Atlantic (mean values) for each species under different climate scenarios.

Species	Area (1000 km^2^)			Range contraction (%)			Minimum latitude of suitability (°N)		
	SSP 1‐1.9	SSP 3‐7.0	SSP 5‐8.5	SSP 1‐1.9	SSP 3‐7.0	SSP 5‐8.5	SSP 1‐1.9	SSP 3‐7.0	SSP 5‐8.5
*Laminaria hyperborea*	186	616	789	12.8	42.3	54.2	41.8	47.4	52.1
*Laminaria digitata*	57	143	225	3.9	9.7	15.3	36.8	42.1	42.7
*Saccharina latissima*	47	122	201	3.4	8.8	14.5	36.9	42.1	42.7
*Alaria esculenta*	272	748	856	17.4	47.7	54.6	43.2	43.2	53.5

**FIGURE A2:**
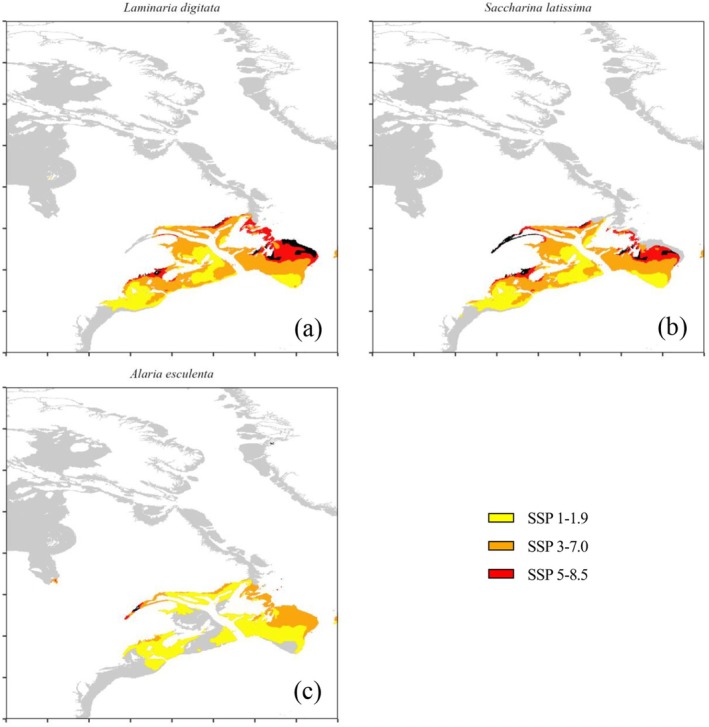
Area lost in the west Atlantic for *Laminaria digitata* (a), *Saccharina latissima* (b), and *Alaria esculenta* (c) in 2100 under SSP1‐1.9 (yellow), SSP3‐7.0 (orange), and SSP5‐8.5 (red). The areas shown for each scenario are cumulative, meaning they include the losses from the previous, less severe scenario(s). Only the additional area lost under a given scenario is shown in its corresponding color. Areas in gray are modeled, but not suitable (see Figure A4). Areas in black are suitable, but not projected to be lost under the climate change scenarios considered.

**FIGURE A3:**
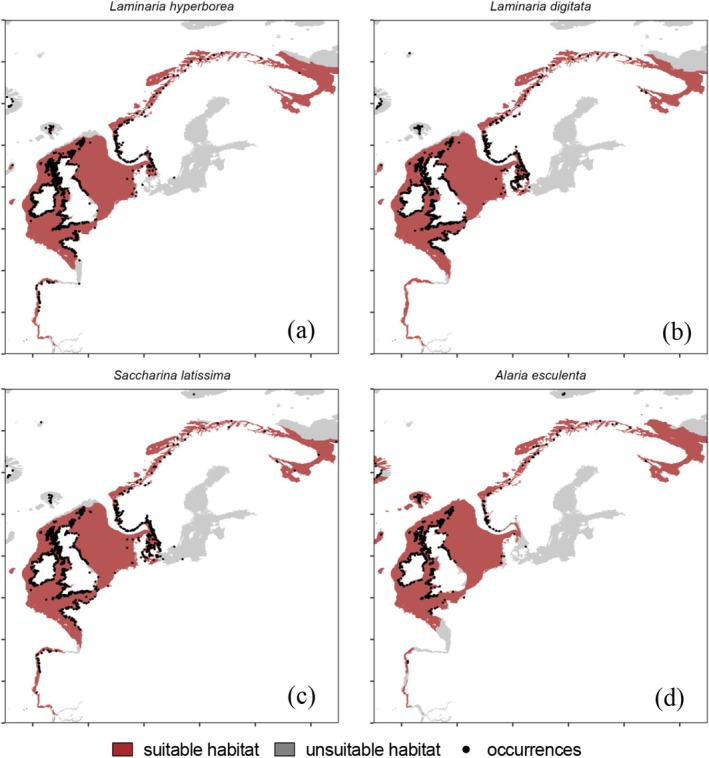
Filtered occurrence points and suitable area in the present East Atlantic post binarization for *Laminaria hyperborea* (a), *Laminaria digitata* (b), *Saccharina latissima* (c), and *Alaria esculenta* (d).

**FIGURE A4:**
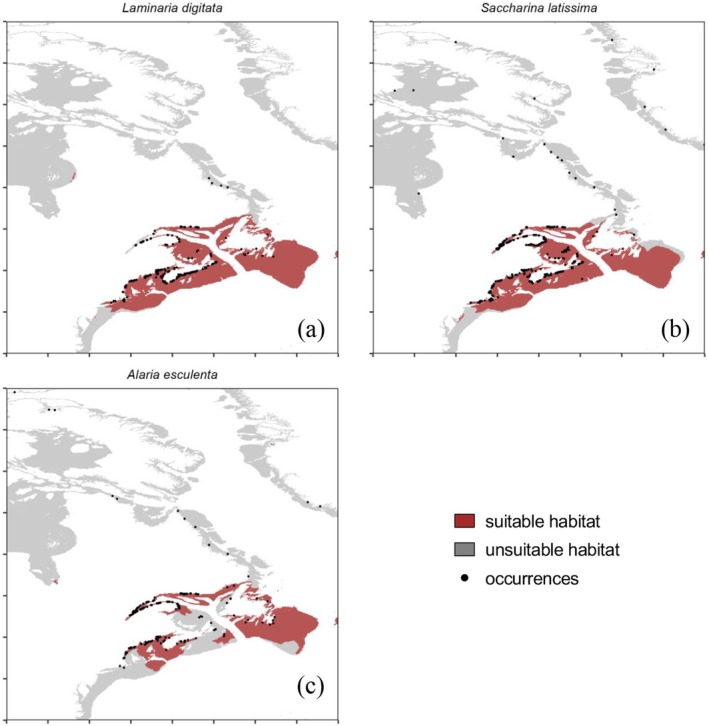
Filtered occurrence points and suitable area in the present West Atlantic post binarization for *Laminaria digitata* (a), *Saccharina latissima* (b), and *Alaria esculenta* (c).

On the west side of the Atlantic, all present kelp species ( *L. hyperborea* does not occur) had projected losses from Cape Cod, Massachusetts, into the Gulf of Maine starting under SSP1‐1.9 (Figure A2). Overall, habitat contraction was higher than on the east side of the Atlantic, exceeding 80% in all species under SSP3‐7.0 (Table 2, Table A2). The minimum latitude of projected suitability (Table 2) was higher under SSP1‐1.9 but lower under SSP5‐8.5 in the West Atlantic, ranging from 40.8° N (New York, *L. digitata* and *S. latissima*, SSP1‐1.9) to 48.0° N (Montréal, *A. esculenta* , SSP5‐8.5).

**TABLE 2:** Area lost (in 1000 km^2^), habitat contraction (in %), and minimum latitude of suitability (°N) in the West Atlantic (mean values) for each species under different climate scenarios.

Species	Area (1000 km^2^)			Range contraction (%)			Minimum latitude of suitability (°N)		
	SSP 1‐1.9	SSP 3‐7.0	SSP 5‐8.5	SSP 1‐1.9	SSP 3‐7.0	SSP 5‐8.5	SSP 1‐1.9	SSP 3‐7.0	SSP 5‐8.5
*Laminaria digitata*	140	398	466	28.3	80.8	94.7	40.8	42.4	44.1
*Saccharina latissima*	153	386	440	32.9	83.1	94.8	40.8	42.4	43.7
*Alaria esculenta*	232	329	331	69.8	98.7	99.4	43.9	47.0	48.0

### 3.3 Area Recovered by Increased Thermal Tolerance

**FIGURE 2:**
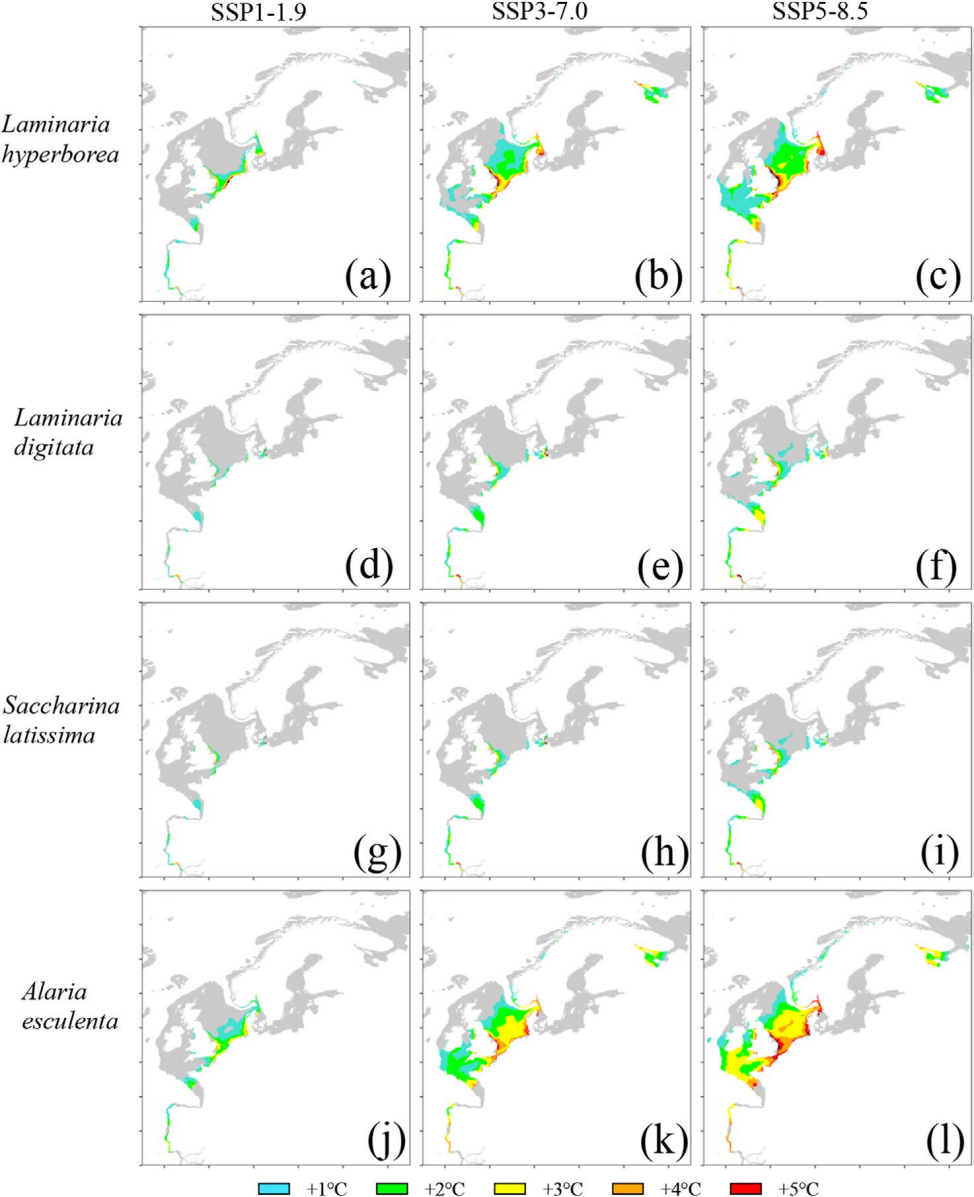
Area recovered in the East Atlantic by *Laminaria hyperborea* (a–c), *Laminaria digitata* (d–f), *Saccharina latissima* (g–i), and *Alaria esculenta* (j–l) under each climate change scenario. Only the additional area recovered under each degree tolerance increase is shown in its corresponding color. Black represents losses not recovered with a 1°C–5°C tolerance increase. Areas in gray are modeled, but either not lost (see Figure A1) or not suitable (see Figure A3).

Under SSP1‐1.9 in the East Atlantic, the most pronounced recovery was in the North Sea for *L. hyperborea* (Figure 2a) and *A. esculenta* (Figure 2j, requiring 3°C–4°C in some areas) while for S. latissima and *L. digitata,* recovery was concentrated in the southeast U.K. (Figure 2d,g). All species had projected patches of recovery on the Iberian coastline in SSP1‐1.9. Under SSP3‐7.0, the North Sea and Skagerrak regions required a tolerance increase of 4°C–5°C in *A. esculenta* (Figure 2k) and *L. hyperborea* (Figure 2b) for complete recovery. *S. latissima* and *L. digitata* recovered the same patches under SSP3‐7.0 with a tolerance increase of 1°C–2°C as they did under SSP1‐1.9, except for the mouth of the Baltic where some areas remained unrecovered, even with a 5°C increase in tolerance (Figure 2e,h). Patches of projected recovery along the Iberian Peninsula became more connected in this scenario and the proportion of this area requiring a tolerance increase greater than 2°C for recovery increased across species. Under SSP5‐8.5, *A. esculenta* required a 4°C tolerance increase in the offshore area of the North Sea for recovery (Figure 2l), but otherwise continued to share large offshore recovered areas with *L. hyperborea*. The Skagerrak region remained a common point of projected losses for all species but required a 5°C tolerance increase for any recovery in *A. esculenta* and *L. hyperborea* , while the other species continued to require increasing tolerance when moving east into the Baltic Sea. The southern edge of the Iberian Peninsula and the neighboring Gibraltar area required a 5°C tolerance increase and continued to host unrecoverable habitat losses under SSP5‐8.5 in all species. Recovery persisted along the remaining western Iberian Peninsula and into Galicia, with a 3°C tolerance increase being required to recover much of Portugal, while 2°C was sufficient in Galicia for all species except *A. esculenta* (Figure 2l).

**FIGURE 3:**
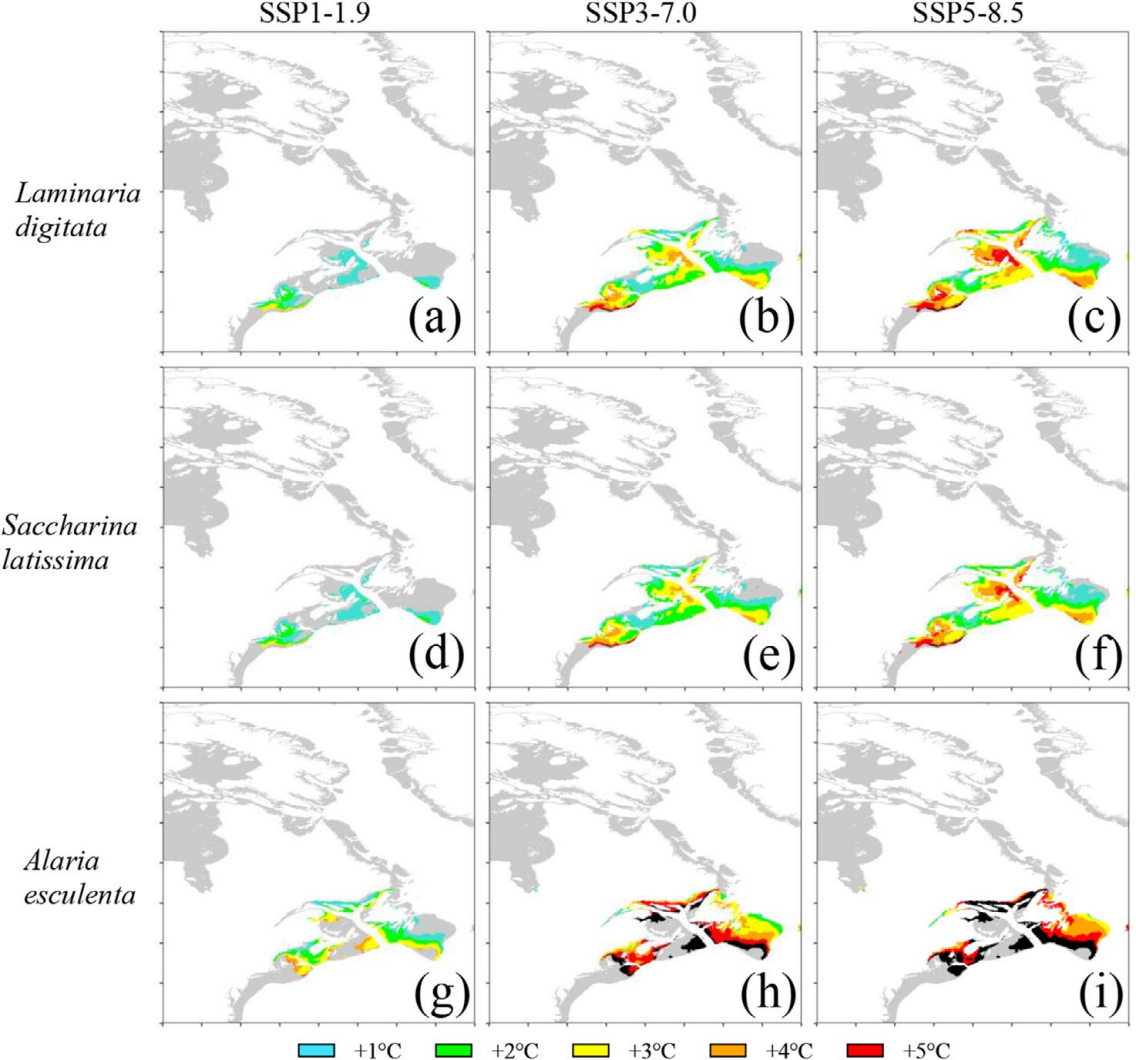
Area recovered in the West Atlantic for *Laminaria digitata* (a–c), *Saccharina latissima* (d–f), and *Alaria esculenta* (g–i) under each climate change scenario. Only the additional area recovered under each degree tolerance increase is shown in its corresponding color. Black represents losses not recovered with a 1°C–5°C tolerance increase. Areas in gray are modeled, but either not lost (see Figure A2) or not suitable (see Figure A4).

In the West Atlantic under SSP1‐1.9, a 3°C tolerance increase was required for recovery at the southern range extent for all species, and in *A. esculenta*, the same amount of tolerance increase was required to recover the Canadian coastline as well (Figure 3). There were no unrecoverable areas in SSP1‐1.9. Under SSP3‐7.0, *A. esculenta* populations became more difficult to recover, with a 4°C–5°C tolerance increase necessary for most of the recovery (Figure 3g). For the other species, the separate recovery “hot spots” of the southern range edge and northeastern Nova Scotia were maintained, requiring a 4°C tolerance increase (5°C on the very southern edge), while the areas in between required less. Under SSP5‐8.5, areas that required a 4°C–5°C tolerance increased and were more prevalent in the West Atlantic compared to the East Atlantic for all species. *A. esculenta* became virtually unrecoverable in coastal areas with anything less than a 5°C tolerance increase, except for small areas of Hudson Bay and the inner St. Lawrence estuary. The focal points of Cape Cod and northeastern Nova Scotia for the other 2 species were firmly out of reach of recovery by any tolerance increase less than 4°C in SSP5‐8.5, though offshore areas and the Bay of Fundy area at the southern end of Nova Scotia remained recoverable with only a 1°C tolerance increase (Figure 3c,f,i).

**FIGURE 4:**
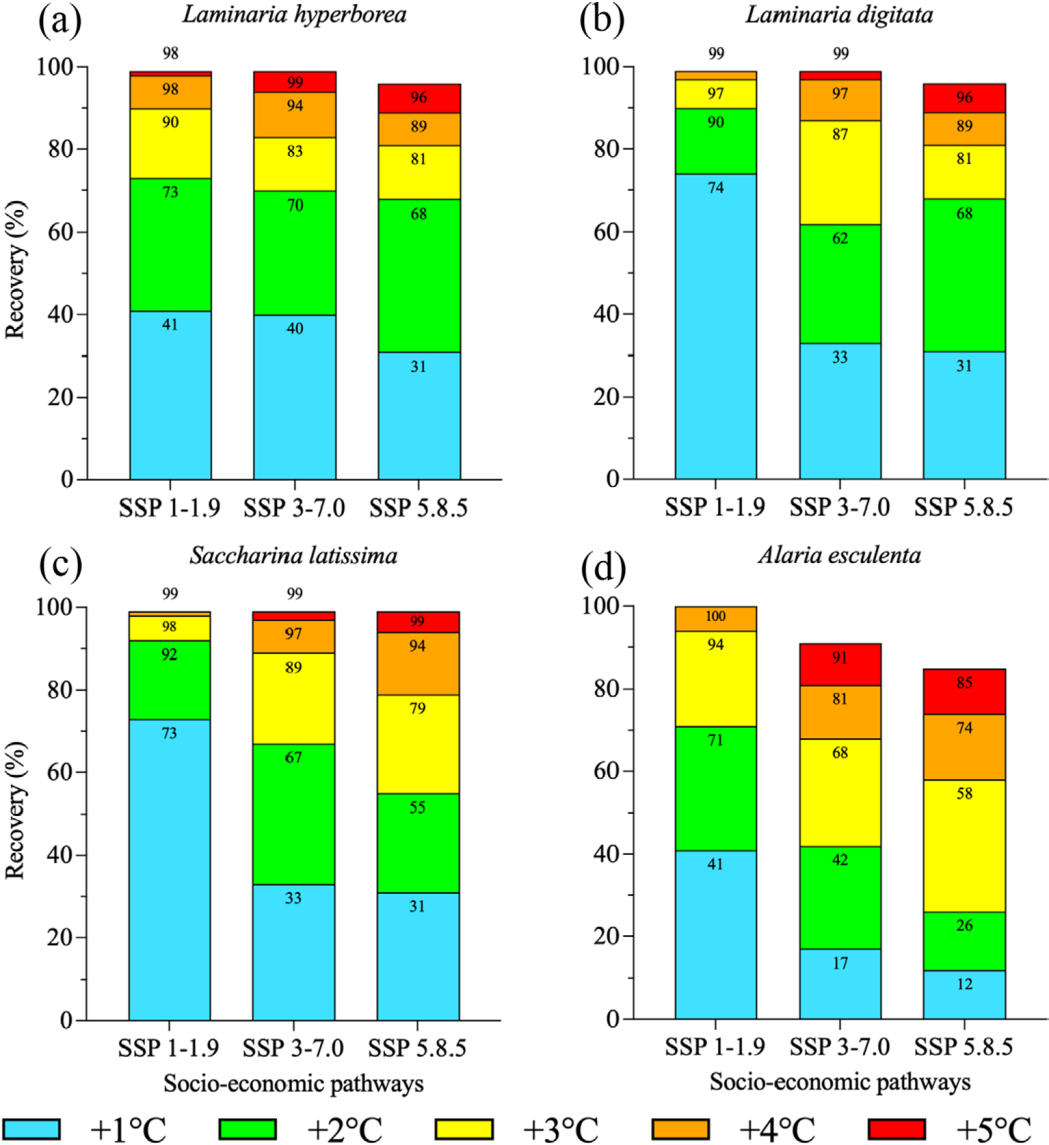
Cumulative percentage of area recovered by degree tolerance increase for *Laminaria hyperborea* (a), *Laminaria digitata* (b), *Saccharina latissima* (c), and *Alaria esculenta* (d) under each scenario.

Overall, *S. latissima* had the highest recovery potential with 99% of its projected lost suitable habitat area recovered under all climate change scenarios, including SSP5‐8.5 (Figure 4c). *L. hyperborea* (Figure 4a) and *L. digitata* (Figure 4b) shared a high recovery rate of 96% with up to 5°C increased upper thermal tolerance under SSP5‐8.5, while *A. esculenta* was the only species with recovery less than 99% under the regional rivalry scenario SSP3‐7.0 (Figure 4d). *A. esculenta* had the lowest overall recovery potential, with 85% recovered, even with a simulated tolerance increase of 5°C in SSP5‐8.5 (Figure 4d), driven primarily by unrecoverable losses in the West Atlantic. All species demonstrated a need for a greater tolerance increase to recover the same habitat percentage as the modeled climate change scenario escalated in severity (Table A3). In 7 out of the 12 species‐scenario combinations, 1°C increased tolerance recovered the largest area; in four combinations, 2°C recovered the most; and only in *A. esculenta* SSP5‐8.5 did 3°C recover the most (Figure 4, Table A3).

## 4 Discussion

### 4.1 Vulnerable Populations and Target Species

Our models of cold‐adapted kelp species across their North Atlantic distribution consistently highlighted temperature as the primary driver influencing their persistence, and all species will face considerable losses in suitable habitat without an increase in temperature tolerance. While most studies tend to focus on the RCP8.5 (corresponding to SSP5‐8.5, Wilson et al. 2019; Assis et al. 2024), our findings indicate that the largest increase in kelp habitat losses occurs between the low‐to moderate‐emission scenarios (SSP1‐1.9 and SSP3‐7.0) rather than between the higher‐emission scenarios (SSP3‐7.0 and SSP5‐8.5). This suggests that significant declines in key kelp populations may arise even under more conservative climate projections, highlighting the urgent need for proactive management strategies.

In the Northwest Atlantic, our projections indicated that the loss of suitable habitat will be greater than 80% for all kelp species in the current study even under the SSP3‐7.0 scenario. While Cape Cod, Massachusetts, has already been recognized as a vulnerable area for *S. latissima* based on physiological thresholds (Wilson et al. 2019), our findings expand the list of at‐risk populations to include Prince Edward's Islands, the West Coast of Newfoundland, and the inner Saint Lawrence region for all modeled kelp species. These projected losses are particularly alarming because the Northwest Atlantic supports some of the world's most valuable kelp forests, which play a crucial role in sustaining invertebrate fisheries, removing nitrogen and carbon (Eger et al. 2023), and underpinning the rapidly expanding seaweed aquaculture sector (Kim et al. 2019). Unlike Europe, which has warm‐temperate kelp species capable of fulfilling similar ecological functions, the Northwest Atlantic lacks such adaptive alternatives that could lead to profound ecosystem disruptions.

Although the situation in the East Atlantic is less severe than in the West Atlantic, our findings still indicate significant habitat losses for all kelp species, even under the most sustainable climate change scenario. For instance, projections of habitat suitability loss under SSP3‐7.0 for *A. esculenta* and *L. hyperborea* in the English Channel and the North Sea align with previous modeling studies conducted under the more extreme RCP8.5 scenario (equivalent to SSP5‐8.5, Assis et al. 2024). Our finding that suitable area persists in northern Norway and the northern U.K. (Figure A1) across climate change scenarios is consistent with the conclusions of multiple studies highlighting the shift in kelp distributions poleward (Raybaud et al. 2013; Assis et al. 2016). Additionally, our analysis identifies new at‐risk regions, including southern and western Norway for *A. esculenta* and *L. hyperborea* as well as the Strait of Dover for *S. latissima* and *L. digitata* , where projected habitat losses have not yet been reported. These findings emphasize the thermal sensitivity of *A. esculenta* and *L. hyperborea* , highlighting the need for prioritizing the conservation of these species.

While the amount of tolerance increase required for recovery is generally highest at lower latitudes, some exceptions exist. Enclosed areas (Skagerrak, English Channel, Gulf of Saint Lawrence, for example) require more of a tolerance increase than expected for their latitude for all modeled kelp species, while upwelling areas (western Iberian coastline) require less of a tolerance increase (Ferreira et al. 2022).

### 4.2 Thermal Tolerance Targets

Across climate change scenarios, we identified 1°C and 2°C as targets of increased thermal tolerance, with an outsized impact, recovering together over 50% of predicted losses on average across species and scenarios. This underscores the importance of applying technologies that aim to enhance the thermal tolerance of these kelp species. Such innovations will be crucial for ensuring the long‐term success of restoration efforts in a changing climate (Coleman et al. 2020).

While 1°C–2°C drives half of all recovery, 3°C and above are required for some of the most productive kelp systems to remain intact. Even under SSP1‐1.9, a 3°C tolerance increase is required to maintain habitat suitability for *A. esculenta* in parts of the Gulf of Maine, Gulf of Saint Lawrence, English Channel, and North Sea. Under SSP3‐7.0, 3°C becomes the minimum requirement to maintain any habitat in these areas for *A. esculenta* and *L. hyperborea* . The Gulf of Maine, Nova Scotia, and the North Sea are experiencing rapid growth in the seaweed aquaculture sector and with ambitious expansion plans in the future that may be jeopardized by the impacts of climate change (Buck et al. 2018; Maar et al. 2023; Kosichek et al. 2024; Brayden and Coleman 2024). While 3°C is a significant demand, given the extent of ecological and economic reliance upon kelp in these areas, it is an important target for initiatives in the coming decades that seek to go beyond the 50% recovery that 1°C–2°C can provide.

### 4.3 Feasibility of Thermal Tolerance Targets

Currently, 1°C–2°C increases in thermal tolerance are possible in kelp via assisted migration (Liesner et al. 2022) and thermal priming (Gauci et al. 2024), suggesting the obstacles to achieving a 2°C target are non‐technical. However, further improvement beyond 2°C may be possible via interventions such as interspecific crosses (Martins et al. 2019) and direct CRISPR‐mediated genetic modification (Shen et al. 2023) but remains the subject of ethical debate. Unfortunately, few macroalgae species have well‐annotated reference genomes available, limiting the application of many of the emerging resilience enhancement mechanisms (Denoeud et al. 2024). In the closely related S*accharina japonica*, the development of strains specifically for cultivation in China began in the 1960s utilizing selective breeding, hybridization, and radiation mutagenesis (Zhang et al. 2011). Continued refinement of the resulting cultivation varieties has produced strains that survive and grow in excess of 20 or even 25°C using only five generations of rigorous selective breeding, without additional genetic information (Liu et al. 2014). The development of a single new strain in 5 years based on cultivars that have been in development for over 5 decades highlights the urgency of developing such approaches in the North Atlantic if useful cultivars are to be developed on a time scale that matches the rate of environmental changes. Our findings suggest that breeding programs and reference genome development for thermally sensitive species such as *A. esculenta* should be a priority if intervention at the level required to meaningfully mitigate losses under SSP3‐7.0 and above is to be possible. While currently unobtainable, the 5°C tolerance increase included as the upper limit in our modified SDMs is shown to be necessary to recover key areas for all species in SSP3‐7.0 and SSP5‐8.5, providing a useful target for future studies and emphasizing that methods to extend tolerance beyond current capabilities are warranted.

### 4.4 Limitations

Despite high model performance, SDMs have limitations that may affect the projected losses and recovery. Binarization of the habitat suitability model output results in a loss of information, especially in marginal habitats, despite being a standard approach to translate raw model output to suitable/non‐suitable predictions (Guillera‐Arroita et al. 2015). Additional limitations arise from projecting any model into the future. The projections used here expand on the relative concentration pathway (RCP) scenarios to include anthropogenic factors such as land‐use changes that influence greenhouse gas emissions (Meinshausen et al. 2020). However, these scenarios represent only one possible interaction between human activity and environmental processes and are inherently uncertain.

Finally, our consideration of modeling areas outside of known distributions to represent possible past and/or future suitable habitat leads to projected suitability where no occurrences are reported, providing an output that corresponds to each kelp's fundamental environmental niche rather than its actual distribution in both the present and future. The model showed a greater tendency to extrapolate suitability to areas without occurrences in the East Atlantic (Iberian Peninsula for *A. esculenta* and *L. digitata* , Gibraltar for all species except *A. esculenta* ). The occurrences with the warmest temperatures in the present were all located along the trailing edge of the North American distribution, suggesting local acclimation that could explain and/or drive the model's projected suitability to latitudes beyond the southern range edge in Europe. These fundamental niches do not reflect other ecological pressures associated with temperature, such as herbivory, that can limit habitat suitability, making higher resolution models or sampling in areas of interest prior to critical to the success of any conservation intervention (Vergés et al. 2014).

## 5 Conclusion

While our results suggest that 1°C–2°C of increased tolerance is sufficient to recover over 50% of projected losses of suitable habitat across species and scenarios, limitations on this recovery as well as on the mechanisms to implement this level of tolerance remain. *A. esculenta* and *L. hyperborea* require additional recovery to maintain their range, making them priority candidates for heat tolerance enhancement. As new resilience enhancement techniques emerge, the thresholds identified here will continue to guide the necessary triage of species and populations in a changing climate. While this study is restricted to the North Atlantic, resilience thresholds are necessary in other warming hotspots such as California and southeast Australia, where kelp losses due to ocean warming are already observed and predicted to accelerate (Wernberg et al. 2018). With minimal modification, similar SDM‐based studies can be conducted for any species and/or region with sufficient occurrence data. It is particularly well‐suited to sessile species including kelp, corals, and seagrasses, as their capacity to relocate in response to climate change is reduced. Overall, the strongest determinant of losses remains climate change scenario, emphasizing that no amount of increased tolerance is a substitute for reductions in greenhouse gas emissions.

## Author Contributions

**Griffin Hill:** conceptualization (lead), data curation (equal), formal analysis (equal), investigation (equal), validation (equal), visualization (equal), writing – original draft (equal), writing – review and editing (equal). **Clément Gauci:** conceptualization (equal), data curation (equal), formal analysis (equal), investigation (equal), methodology (equal), validation (equal), visualization (equal), writing – original draft (equal), writing – review and editing (equal). **Jorge Assis:** conceptualization (supporting), data curation (equal), formal analysis (supporting), investigation (supporting), methodology (supporting), resources (equal), validation (equal), visualization (supporting), writing – review and editing (equal). **Alexander Jueterbock:** conceptualization (supporting), data curation (supporting), formal analysis (supporting), funding acquisition (equal), investigation (supporting), methodology (supporting), project administration (equal), resources (equal), supervision (equal), validation (equal), visualization (supporting), writing – review and editing (equal).

## Conflicts of Interest

The authors declare no conflicts of interest.

## Data Availability

All data, scripts, and files required to reproduce these analyses at the time of publication are publicly available in a Data Dryad repository at https://doi.org/10.5061/dryad.f4qrfj751.

## Appendix A

**TABLE A1:** Model evaluation and variable contribution (%) for each increased heat tolerance and species.

Species	Increased tolerance	AUC	TSS	Suitability peak	Max SST (%)	Mean phosphate (%)	Mean salinity (%)	Mean nitrate (%)
*Laminaria hyperborea*	Base model	0.90	0.65	14.82	46.74 ± 3.07	35.08 ± 2.49	18.18 ± 1.31	
	1°C	0.90	0.66	15.79	49.52 ± 3.31	34.08 ± 2.59	16.44 ± 1.22	
	2°C	0.90	0.67	16.39	50.88 ± 3.58	33.64 ± 2.66	15.46 ± 1.31	
	3°C	0.90	0.67	16.93	51.74 ± 3.79	33.40 ± 2.74	14.86 ± 1.40	
	4°C	0.91	0.68	17.48	52.30 ± 3.98	33.26 ± 2.83	14.46 ± 1.47	
	5°C	0.91	0.68	18.02	52.64 ± 4.13	33.16 ± 2.89	14.20 ± 1.50	
*Laminaria digitata*	Base model	0.87	0.60	15.78	54.16 ± 5.14	27.08 ± 4.43	18.76 ± 1.57	
	1°C	0.88	0.61	16.53	54.10 ± 5.13	27.06 ± 4.49	18.82 ± 1.53	
	2°C	0.88	0.61	17.17	53.90 ± 5.04	27.22 ± 4.47	18.84 ± 1.48	
	3°C	0.88	0.61	17.81	53.70 ± 4.95	27.42 ± 4.44	18.86 ± 1.47	
	4°C	0.88	0.62	18.46	53.54 ± 4.86	27.58 ± 4.43	18.90 ± 1.47	
	5°C	0.89	0.62	19.04	53.34 ± 4.76	27.78 ± 4.38	18.90 ± 1.48	
*Saccharina latissima*	Base model	0.86	0.59	17.34	49.74 ± 4.19	26.44 ± 3.22	18.62 ± 1.31	5.22 ± 1.62
	1°C	0.87	0.59	17.78	48.16 ± 3.72	27.86 ± 2.88	19.08 ± 1.35	4.84 ± 1.57
	2°C	0.87	0.60	18.25	47.26 ± 3.58	28.60 ± 2.80	19.30 ± 1.34	4.78 ± 1.52
	3°C	0.87	0.60	18.88	46.56 ± 3.53	29.20 ± 2.74	19.44 ± 1.37	4.78 ± 1.45
	4°C	0.87	0.60	19.51	45.84 ± 3.48	29.76 ± 2.68	19.62 ± 1.33	4.80 ± 1.45
	5°C	0.87	0.60	20.14	45.08 ± 3.50	30.32 ± 2.69	19.78 ± 1.36	4.82 ± 1.45
*Alaria esculenta*	Base model	0.87	0.57	14.86	56.18 ± 5.55	19.86 ± 2.12	13.74 ± 1.89	10.24 ± 3.43
	1°C	0.87	0.59	15.67	57.68 ± 5.72	18.98 ± 2.08	13.24 ± 1.76	10.12 ± 3.28
	2°C	0.87	0.60	16.19	58.62 ± 5.80	18.44 ± 2.13	12.88 ± 1.70	10.06 ± 3.14
	3°C	0.87	0.60	16.71	59.38 ± 5.81	18.08 ± 2.12	12.58 ± 1.66	9.94 ± 3.08
	4°C	0.87	0.60	17.57	59.88 ± 5.78	17.78 ± 2.07	12.42 ± 1.62	9.88 ± 2.96
	5°C	0.87	0.60	18.13	60.20 ± 5.78	17.56 ± 2.03	12.36 ± 1.54	9.88 ± 2.90

**TABLE A2:** Area lost per species and scenario (overall).

Species	Area (km^−2^)			Habitat contraction (%)			Minimum latitude of suitability (°N)		
	SSP 1‐1.9	SSP 3‐7.0	SSP 5‐8.5	SSP 1‐1.9	SSP 3‐7.0	SSP 5‐8.5	SSP 1‐1.9	SSP 3‐7.0	SSP 5‐8.5
*Laminaria hyperborea*	186,283	616,153	789,083	12.8	42.3	54.2	41.8	47.4	52.1
*Laminaria digitata*	196,974	540,918	691,961	10.0	27.5	35.2	36.8	42.1	42.7
*Saccharina latissima*	199,901	508,304	641,176	10.8	27.5	34.7	36.9	42.1	42.7
*Alaria esculenta*	504,718	1,076,859	1,187,110	26.5	56.6	62.4	43.2	43.2	53.5

**TABLE A3:** Area recovered in 2100 by each increase in heat tolerance for each species and climate scenario.

Species	Increased tolerance	Area (km^2^)			Area (%)		
		SSP 1‐1.9	SSP 3‐7.0	SSP 5‐8.5	SSP 1‐1.9	SSP 3‐7.0	SSP 5‐8.5
*Laminaria hyperborea*	+1°C	113,134	279,739	272,632	41	40	31
	+2°C	200,185	491,769	596,476	73	69	68
	+3°C	246,768	581,382	708,569	90	82	80
	+4°C	268,507	655,979	781,824	98	93	89
	+5°C	272,102	693,294	846,407	99	98	96
*Laminaria digitata*	+1°C	146,014	178,276	214,920	74	33	31
	+2°C	178,235	333,265	357,436	90	62	52
	+3°C	192,366	469,036	521,876	98	87	75
	+4°C	195,455	525,357	635,272	99	97	92
	+5°C	195,254	537,428	686,249	99	99	99
*Saccharina latissima*	+1°C	145,648	168,881	201,214	73	33	31
	+2°C	183,976	344,232	355,031	92	68	55
	+3°C	195,001	455,326	508,524	98	90	79
	+4°C	197,766	497,649	607,786	99	98	95
	+5°C	197,670	505,815	638,109	99	100	100
*Alaria esculenta*	+1°C	204,757	181,036	143,715	41	17	12
	+2°C	357,658	455,473	313,910	71	42	26
	+3°C	474,601	738,993	690,182	94	69	58
	+4°C	503,817	882,680	880,983	100	82	74
	+5°C	504,718	990,250	1,009,456	100	92	85

## Appendix B

In the East Atlantic, *A. esculenta* had the highest projected losses spanning most of the Brittany coast, English Channel, and the North Sea coast. Offshore, its projected losses overlapped largely with those of *L. hyperborea* in the North Sea and around the southern U.K. The Skagerrak region had projected losses for all species beginning in SSP1‐1.9 (Figure A1). *S. latissima* and *L. digitata* had projected losses that extended from the Skagerrak into the mouth of the Baltic Sea beginning in SSP1‐1.9 as well. At the southern extent of the species ranges, losses in SSP1‐1.9 began around the Strait of Gibraltar and extended northward to varying degrees, reaching as far as Galicia, Spain depending on the species. Galicia stands out as the region with the greatest variation in projected losses across species and climate scenarios. *S. latissima* and *L. digitata* were projected to face complete habitat loss in Galicia only under SSP5‐8.5. In contrast, *L. hyperborea* was projected to disappear from the area under SSP3‐7.0, and *A. esculenta* was already under the mildest scenario SSP1‐1.9.

In the West Atlantic, *L. digitata* and *S. latissima* also were projected to suffer losses around Nova Scotia and in the Gulf of Saint Lawrence starting under SSP1‐1.9 (totaling 28.3% and 32.9% habitat contraction respectively, with a minimum latitude of projected suitability at 40.8° N for both species, Table A2). *A. esculenta* hosted a smaller suitable habitat area in the present in North America and was projected to lose almost all of it: (69.8% projected habitat contraction under SSP1‐1.9 with a minimum latitude of projected suitability at 43.9° N, and 98.7% contraction and with 47.0° N under SSP3‐7.0, Table A2 and Figure A2). *L. digitata* and *S. latissima* had nearly identical projected losses under SSP3‐7.0 (80.8% and 83.1%, respectively, with a minimum latitude of projected suitability at 42.4° N) and SSP5‐8.5 (94.7% and 94.8%, 44.1° N and 43.7° N, respectively), expanding to Prince Edward Island and the offshore areas on either side of the Laurentian Channel (Figure A2). *A. esculenta* was projected to lose 95.8% of its suitable habitat under SSP3‐7.0, with the small remnant areas persisting in Hudson Bay and the St. Lawrence estuary being lost under SSP5‐8.5, bringing total projected losses in the western Atlantic to 99.4%, and a minimum latitude of projected suitability at 48° N (completely above Maine).

## References

[ece371271-bib-0001] Allouche, O. , A. Tsoar , and R. Kadmon . 2006. “Assessing the Accuracy of Species Distribution Models: Prevalence, Kappa and the True Skill Statistic (TSS).” Journal of Applied Ecology 43: 1223–1232. 10.1111/j.1365-2664.2006.01214.x.

[ece371271-bib-0002] Araújo, R. M. , J. Assis , R. Aguillar , et al. 2016. “Status, Trends and Drivers of Kelp Forests in Europe: An Expert Assessment.” Biodiversity and Conservation 25: 1319–1348. 10.1007/s10531-016-1141-7.

[ece371271-bib-0003] Assis, J. , E. Fragkopoulou , D. Frade , et al. 2020. “A Fine‐Tuned Global Distribution Dataset of Marine Forests.” Scientific Data 7: 119. 10.1038/s41597-020-0459-x. 32286314 PMC7156423

[ece371271-bib-0004] Assis, J. , E. Fragkopoulou , L. Gouvêa , M. B. Araújo , and E. A. Serrão . 2024. “Kelp Forest Diversity Under Projected End‐Of‐Century Climate Change.” Diversity and Distributions 30: e13837. 10.1111/ddi.13837.

[ece371271-bib-0005] Assis, J. , A. V. Lucas , I. Bárbara , and E. Á. Serrão . 2016. “Future Climate Change Is Predicted to Shift Long‐Term Persistence Zones in the Cold‐Temperate Kelp *Laminaria hyperborea* .” Marine Environmental Research 113: 174–182. 10.1016/j.marenvres.2015.11.005. 26608411

[ece371271-bib-0006] Barrientos, S. , C. Piñeiro‐Corbeira , and R. Barreiro . 2025. “Twenty‐Five Years on: Widespread Kelp Forest Decline Revealed in a Potential Climatic Refugium.” Journal of Environmental Management 373: 123734. 10.1016/j.jenvman.2024.123734. 39700941

[ece371271-bib-0007] Brayden, C. , and S. Coleman . 2024. “Maine Seaweed Benchmarking: Economically Assessing the Growth of an Emerging Sector.” Aquaculture Economics & Management 28: 491–514. 10.1080/13657305.2024.2319086.

[ece371271-bib-0008] Buck, B. H. , M. F. Troell , G. Krause , D. L. Angel , B. Grote , and T. Chopin . 2018. “State of the Art and Challenges for Offshore Integrated Multi‐Trophic Aquaculture (IMTA).” Frontiers in Marine Science 5: 165. 10.3389/fmars.2018.00165.

[ece371271-bib-0009] Coleman, M. A. , G. Wood , K. Filbee‐Dexter , et al. 2020. “Restore or Redefine: Future Trajectories for Restoration.” Frontiers in Marine Science 7: 237. 10.3389/fmars.2020.00237.

[ece371271-bib-0047] Denoeud, F. , O. Godfroy , C. Cruaud , et al. 2024. “Evolutionary Genomics of the Emergence of Brown Algae as Key Components of Coastal Ecosystems.” Cell 187, no. 24: 6943–6965.e39. 10.1016/j.cell.2024.10.049. 39571576

[ece371271-bib-0011] Eger, A. M. , E. M. Marzinelli , R. Beas‐Luna , et al. 2023. “The Value of Ecosystem Services in Global Marine Kelp Forests.” Nature Communications 14: 1894. 10.1038/s41467-023-37385-0. PMC10113392 37072389

[ece371271-bib-0012] Eger, A. M. , E. M. Marzinelli , H. Christie , et al. 2022. “Global Kelp Forest Restoration: Past Lessons, Present Status, and Future Directions.” Biological Reviews 97: 1449–1475. 10.1111/brv.12850. 35255531 PMC9543053

[ece371271-bib-0013] Ferreira, S. , M. Sousa , A. Picado , N. Vaz , and J. M. Dias . 2022. “New Insights About Upwelling Trends off the Portuguese Coast: An ERA5 Dataset Analysis.” Journal of Marine Science and Engineering 10: 1849. 10.3390/jmse10121849.

[ece371271-bib-0014] Filbee‐Dexter, K. , T. Wernberg , R. Barreiro , et al. 2022. “Leveraging the Blue Economy to Transform Marine Forest Restoration.” Journal of Phycology 58: 198–207. 10.1111/jpy.13239. 35092031

[ece371271-bib-0015] Filbee‐Dexter, K. , T. Wernberg , S. P. Grace , et al. 2020. “Marine Heatwaves and the Collapse of Marginal North Atlantic Kelp Forests.” Scientific Reports 10: 13388. 10.1038/s41598-020-70273-x. 32770015 PMC7414212

[ece371271-bib-0016] Fragkopoulou, E. , E. A. Serrão , O. De Clerck , et al. 2022. “Global Biodiversity Patterns of Marine Forests of Brown Macroalgae.” Global Ecology and Biogeography 31: 636–648. 10.1111/geb.13450.

[ece371271-bib-0018] Gauci, C. , A. Jueterbock , A. Khatei , G. Hoarau , and I. Bartsch . 2024. “Thermal Priming of Saccharina Latissima: A Promising Strategy to Improve Seaweed Production and Restoration in Future Climates.” Marine Ecology Progress Series 745: 59–71. 10.3354/meps14683.

[ece371271-bib-0019] Guillera‐Arroita, G. , J. J. Lahoz‐Monfort , J. Elith , et al. 2015. “Is My Species Distribution Model Fit for Purpose? Matching Data and Models to Applications.” Global Ecology and Biogeography 24: 276–292. 10.1111/geb.12268.

[ece371271-bib-0020] Guiry, M. D. , and G. M. Guiry . 2024. AlgaeBase.

[ece371271-bib-0046] Gurgel, C. F. D. , O. Camacho , A. J. P. Minne , T. Wernberg , and M. A. Coleman . 2020. “Marine Heatwave Drives Cryptic Loss of Genetic Diversity in Underwater Forests.” Current Biology 30, no. 7: 1199–1206.e2. 10.1016/j.cub.2020.01.051. 32109397

[ece371271-bib-0021] Hiscock, K. , and H. Tyler‐Walters . 2006. “Assessing the Sensitivity of Seabed Species and Biotopes – The Marine Life Information Network (MarLIN).” Hydrobiologia 555: 309–320. 10.1007/s10750-005-1127-z.

[ece371271-bib-0022] Jueterbock, A. , A. J. P. Minne , J. M. Cock , et al. 2021. “Priming of Marine Macrophytes for Enhanced Restoration Success and Food Security in Future Oceans.” Frontiers in Marine Science 8: 658485. 10.3389/fmars.2021.658485.

[ece371271-bib-0023] Kim, J. , M. Stekoll , and C. Yarish . 2019. “Opportunities, Challenges and Future Directions of Open‐Water Seaweed Aquaculture in the United States.” Phycologia 58: 446–461. 10.1080/00318884.2019.1625611.

[ece371271-bib-0024] Kosichek, H. , J. Reimer , and R. Filgueira . 2024. “Assessing the Potential for Seaweed Aquaculture in Nova Scotia.” Aquaculture Reports 36: 102064. 10.1016/j.aqrep.2024.102064.

[ece371271-bib-0025] Landis, J. R. , and G. G. Koch . 1977. “The Measurement of Observer Agreement for Categorical Data.” Biometrics 33: 159–174. 10.2307/2529310. 843571

[ece371271-bib-0026] Liesner, D. , G. A. Pearson , I. Bartsch , et al. 2022. “Increased Heat Resilience of Intraspecific Outbred Compared to Inbred Lineages in the Kelp *Laminaria digitata* : Physiology and Transcriptomics.” Frontiers in Marine Science 9: 838793. 10.3389/fmars.2022.838793.

[ece371271-bib-0027] Liu, F. , X. Sun , F. Wang , et al. 2014. “Breeding, Economic Traits Evaluation, and Commercial Cultivation of a New Saccharina Variety “Huangguan No. 1”.” Aquaculture International 22: 1665–1675. 10.1007/s10499-014-9772-8.

[ece371271-bib-0028] Maar, M. , A. Holbach , T. Boderskov , et al. 2023. “Multi‐Use of Offshore Wind Farms With Low‐Trophic Aquaculture Can Help Achieve Global Sustainability Goals.” Communications Earth & Environment 4: 1–14. 10.1038/s43247-023-01116-6.37325084

[ece371271-bib-0029] Mann, K. 1973. “Seaweeds: Their Productivity and Strategy for Growth.” Science 182: 975–981. 10.1126/science.182.4116.975. 17833778

[ece371271-bib-0030] Martins, N. , G. A. Pearson , L. Gouveia , A. I. Tavares , E. A. Serrão , and I. Bartsch . 2019. “Hybrid Vigour for Thermal Tolerance in Hybrids Between the Allopatric Kelps *Laminaria digitata* and *L. pallida* (Laminariales, Phaeophyceae) With Contrasting Thermal Affinities.” European Journal of Phycology 54: 548–561. 10.1080/09670262.2019.1613571.

[ece371271-bib-0031] Meinshausen, M. , Z. R. J. Nicholls , J. Lewis , et al. 2020. “The Shared Socio‐Economic Pathway (SSP) Greenhouse Gas Concentrations and Their Extensions to 2500.” Geoscientific Model Development 13: 3571–3605. 10.5194/gmd-13-3571-2020.

[ece371271-bib-0032] Muth, A. F. , M. H. Graham , C. E. Lane , and C. D. G. Harley . 2019. “Recruitment Tolerance to Increased Temperature Present Across Multiple Kelp Clades.” Ecology 100: e02594. 10.1002/ecy.2594. 30615200

[ece371271-bib-0033] Ocean Biodiversity Information System . 2024. “WWW Document.” Accessed October 2, 2025. https://www.obis.org/.

[ece371271-bib-0034] Phillips, S. J. , and M. Dudík . 2008. “Modeling of Species Distributions With Maxent: New Extensions and a Comprehensive Evaluation.” Ecography 31: 161–175. 10.1111/j.0906-7590.2008.5203.x.

[ece371271-bib-0035] Provoost, P. , and S. Bosch . 2017. “robis: R Client to Access Data From the OBIS API. Ocean Biogeographic Information System. Intergovernmental Oceanographic Commission of UNESCO.”

[ece371271-bib-0036] Raybaud, V. , G. Beaugrand , E. Goberville , et al. 2013. “Decline in Kelp in West Europe and Climate.” PLoS One 8: e66044. 10.1371/journal.pone.0066044. 23840397 PMC3694085

[ece371271-bib-0037] Shen, Y. , T. Motomura , K. Ichihara , et al. 2023. “Application of CRISPR‐Cas9 Genome Editing by Microinjection of Gametophytes of Saccharina Japonica (Laminariales, Phaeophyceae).” Journal of Applied Phycology 35: 1431–1441. 10.1007/s10811-023-02940-1.

[ece371271-bib-0038] Teagle, H. , S. J. Hawkins , P. J. Moore , and D. A. Smale . 2017. “The Role of Kelp Species as Biogenic Habitat Formers in Coastal Marine Ecosystems.” Journal of Experimental Marine Biology and Ecology Ecological responses to environmental change in marine systems 492: 81–98. 10.1016/j.jembe.2017.01.017.

[ece371271-bib-0039] Tullberg, R. M. , H. P. Nguyen , and C. M. Wang . 2022. “Review of the Status and Developments in Seaweed Farming Infrastructure.” Journal of Marine Science and Engineering 10: 1447. 10.3390/jmse10101447.

[ece371271-bib-0040] Valavi, R. , J. Elith , J. J. Lahoz‐Monfort , and G. Guillera‐Arroita . 2019. “blockCV: An r Package for Generating Spatially or Environmentally Separated Folds for k‐Fold Cross‐Validation of Species Distribution Models.” Methods in Ecology and Evolution 10: 225–232. 10.1111/2041-210X.13107.

[ece371271-bib-0041] Vergés, A. , P. D. Steinberg , M. E. Hay , et al. 2014. “The Tropicalization of Temperate Marine Ecosystems: Climate‐Mediated Changes in Herbivory and Community Phase Shifts.” Proceedings of the Royal Society B: Biological Sciences 281, no. 1789: 20140846. 10.1098/rspb.2014.0846. PMC4100510 25009065

[ece371271-bib-0042] Wernberg, T. , M. A. Coleman , S. Bennett , M. S. Thomsen , F. Tuya , and B. P. Kelaher . 2018. “Genetic Diversity and Kelp Forest Vulnerability to Climatic Stress.” Scientific Reports 8: 1851. 10.1038/s41598-018-20009-9. 29382916 PMC5790012

[ece371271-bib-0043] Wilson, K. L. , M. A. Skinner , and H. K. Lotze . 2019. “Projected 21st‐Century Distribution of Canopy‐Forming Seaweeds in the Northwest Atlantic With Climate Change.” Diversity and Distributions 25: 582–602. 10.1111/ddi.12897.

[ece371271-bib-0044] Zhang, J. , Y. Liu , D. Yu , H. Song , J. Cui , and T. Liu . 2011. “Study on High‐Temperature‐Resistant and High‐Yield Laminaria Variety “Rongfu”.” Journal of Applied Phycology 23: 165–171. 10.1007/s10811-011-9650-y.

[ece371271-bib-0045] Zhou, R. , F. Jiang , L. Niu , et al. 2022. “Increase Crop Resilience to Heat Stress Using Omic Strategies.” Frontiers in Plant Science 13: 891861. 10.3389/fpls.2022.891861. 35656008 PMC9152541

